# Frontiers in Vascular Physiology Grand Challenges in Vascular Physiology

**DOI:** 10.3389/fphys.2020.00852

**Published:** 2020-08-07

**Authors:** Gerald A. Meininger, Michael A. Hill

**Affiliations:** Dalton Cardiovascular Research Center and Department of Medical Pharmacology and Physiology, University of Missouri, Columbia, MO, United States

**Keywords:** vascular, cardiovascular, medical, vascular physiology, circulation

We have reached a new decade and have witnessed a plethora of remarkable advances in biology and medicine over the last decade. Importantly, the 2020's certainly promise to deliver even more exciting innovations than the last decade as we continue to integrate molecular and cellular data into a more comprehensive understanding at the tissue, organ, system, and organismal levels. For vascular physiology and medicine these are especially exciting times. We have witnessed rapid advances in vascular biology-related genetic biology, technological advancements in the bioengineering of organs and tissue, improvements in instrumentation and technology, progress in robotics, and artificial intelligence to name but a few. The principal objective of this paper is to highlight key recent achievements, future possibilities and the need for continued advances in the field of vascular physiology.

We have a continuing need for investment of resources in research directed at cardiovascular disease (CVD). As a non-communicable disease, morbidity and mortality from cardiovascular pathology is one of the major health issues that impacts the quality of life and contributes to premature death. Despite a reduction in CVD related mortality as a consequence of developments in both medical and surgical interventions (Mensah et al., 2017), this remains a worldwide health problem with immense economic impact. Using data provided by the American Heart Association for the United States as an example, the cost of CVD and stroke was estimated at $555 billion for 2016 while being projected to rise to over $1 trillion by 2035 (American Heart Association, 2017; Benjamin et al., 2019). These figures rise considerably if costs related to lost productivity and nursing homes are estimated. Impact fact statements for the UK (UK Fact Sheet, 2019) estimating yearly costs of CVD at 19 billion GBP and multiple billions of Euros across Europe (Timmis et al., 2020) are a further testimony to the negative effects of CVD on our society. Almost certainly similar data exists globally, particularly with the “westernization” of developing countries. These are staggering and sobering figures that underscore the need for continued research into the risk factors that predispose toward developing CVD and into the mechanistic causes and therapeutic approaches to treatment and management. Based on this economic and health related data it is understandable why there has been a shift toward translational/clinical relevance however, this must not exclude fundamental research in cardiovascular physiology which remains an essential approach to understand important disease processes.

In the coming decade will see increasing challenges in the cardiovascular health field, particularly as rates of insulin resistance, obesity and overt type II diabetes continue to rise (Ward et al., 2019). In this regard, in the United States, obesity (BMI > 30) and severe obesity (BMI > 35) are predicted to reach ~50 and 25% of the population, respectively, by 2030 (Ward et al., 2019). Similarly, the cardiovascular impact of exposure to particulate matter is being more and more appreciated. Increasing evidence is accumulating that exposure to airborne inhalable (PM_10_) and fine (PM_2.5_) particles predispose to cardiovascular disorders including those that are independent of pulmonary effects (Hamanaka and Mutlu, 2018; Zhang et al., 2018; Combes and Franchineau, 2019). Thus, exposure to particulate matter has been linked to increased prevalence of metabolic disorders and even to predisposing the developing fetus to vascular disease in later life. Recently, the cardiovascular impacts of electronic cigarettes, or “vaping” is also now on the horizon. Further, cardiovascular disorders have emerged as a result of long-term medical treatments (e.g., cardiotoxicity of anti-cancer agents) and increased longevity in patients with chronic conditions (e.g., Duchenne's Muscular Dystrophy and cardiomyopathy). As we have realized over the last decade, there is an increasing rate of CVD driven by the Westernization of developing countries. As mentioned above, this trend continues. Finally, the current SARS-CoV2 (Covid-19) pandemic highlights the multiple interactions that can occur between an infectious viral disease and the cardiovascular system. Although early in our full understanding of the pathogenesis of Covid-19 infection, the cardiovascular system contributes to cellular viral entry via the angiotensin converting enzyme 2 (ACE2) (Wan et al., 2020) while also being directly impacted by the virus (for example as reflected in myocarditis and clotting abnormalities) (Libby, 2020; Spiezia et al., 2020). Further, severity of Covid-19 infection, in terms of negative outcomes, is negatively affected by multiple factors impacting the cardiovascular system including co-existing obesity, cardiometabolic disease, diabetes and health disparities (Belanger et al., 2020; Hill et al., 2020; Muniyappa and Gubbi, 2020; Wu and McGoogan, 2020).

These CVD statistics are certainly dramatic but looking forward there are many ongoing developments in the field that will have significant impacts on cardiovascular research and health over the next decade. Imaging continues to be a technology-based shining star. Consistent with the adage, “if you can see it you can study it,” new developments in imaging are continuing to emerge. In the world of optical microscopy we are seeing improvements in resolution that are allowing visualization of molecular events, new fluorescent agents are improving selective targeting and microscope automation is driving large increases in throughput. Such improved modes of imaging include super resolution, light sheet microscopy with 4D capability as well as increased application of optical techniques such as cryo-electron microscopy. The increased availability of these approaches will benefit the cardiovascular field. In the last decade we have also witnessed a rapid growth in the use and application development of techniques involving scanning probe technology, for example, atomic force microscopy. These techniques have proven their usefulness in allowing single molecular events to be studied and to biomechanically probe cells and molecules. Rapid advances in high speed atomic force microscopy have advanced this technology even further into the realm of observing of molecular events and it will be exciting to see further developments allowing reliable live cell applications to become a reality. In cardiovascular research we need further development of applications to permit visualization of the vasculature at all scales of organization from large conduit vessels to the smallest vessels of the microcirculation. Techniques for measurements of vascular dimensions, perfusion, circulating cell distribution, and vessel wall biomechanics to name a few are all critical parameters for the study of organ function in health and disease. As the endeavors of developmental biology, regenerative medicine, organ transplantation, and artificial organs advance the ability to monitor the functioning vasculature is key for the success of these endeavors.

Over the coming decade there will be an increased availability of human data through electronic medical records and commercial sources. Tissue obtainable through biorepositories, increased access to patient databases and genetic screening, non-invasive ambulatory technologies that monitor health, drug delivery and communicate with doctors are all examples of new emerging sources of material for research. With all of these technologies and the automation in computer driven data collection there has been a tremendous increase in the size of data sets. Multi megabyte and even terabyte data sets are becoming more common, the magnitude of which is beyond human capacity to critically evaluate such results. Thus, there is a critical demand for improvements in bioinformatics and data processing that will ease user interaction with these large data sets and permit the objectivity needed to extract essential features for accurate hypothesis testing.

Over the last decade there has been a continued emphasis on reductionism as we strive to understand biological function even more deeply. As we look forward at ways to interpret the relevance of this information there is a necessity for continued development of non-invasive and minimally invasive forms of imaging and biosensing that will allow study of physiological parameters and biochemical events in intact animals and subjects. In this regard, a noticeable shift in research strategy during the last decade has been to complement cellular and molecular approaches with sophisticated *in vivo* techniques. Of particular significance are approaches allowing temporal analysis of disease models as provided by imaging techniques (MRI, SPECT, high frequency ultrasound and photoacoustics, telemetry), metabolic phenotyping, and behavioral analysis. These are very encouraging developments that will help to promote a more integrative view and improved accuracy of interpretation culminating in a more integrative approach to understanding cell molecular data. Physiology is, after all, a field that strives to understand the intact functioning system at the organ and organismal level. It equally important we see these trends extended to an educational level where a resurgence of some classical discipline-based training is important and should be given increased priority. The growing need for research using whole animal and organ level experimental research design requires an increasing not shrinking population of physiologists with the skills to perform these types of experiments.

Another area that should not be overlooked in the coming decade is continuing to build our skills in modeling biological systems. The ability to produce accurate mathematical models of complex data and complex system behavior is proving an important partner in hypothesis generation and testing and data interpretation. Models provide the ability to produce physical representations underlying experimental results. This allows novel insight into data and provides a means for generating testable new hypotheses. Some examples include, understanding perfusion patterns, nutrient distribution, distribution of mechanical forces within the vascular system, vascular remodeling and development, drug delivery and targeting, and molecular behavior underlying cell and vessel scale behaviors. Thus, modeling contributes greatly to our understanding of cardiac and vascular function by significantly expanding the number of potential interacting variables that can be investigated and dynamically tracked. Searching for single variable answers in complex and dynamic systems is often a flawed approach to the complex hypotheses we are attempting to test. Further, the availability of approaches for handling large data sets will be critical for the development and implementation of powerful machine learning approaches for the study of complex cellular signaling networks relevant to cardiovascular tissue and disease processes (Ping et al., 2018).

Although the next decade looks extremely promising we have unfortunately seen the last decade present some real threats to the quality of cardiovascular research and biomedical research, in general. Specifically, it is worth mentioning that as scientists we are facing a time when we have to exercise increased ethical vigilance when we select journals to publish in and scientific meetings to attend. The integrity of the field is now impacted by the proliferation of journals that seem to be driven by profit rather than the science. Many of these have been referred to as “predatory journals” as typified by Beal's List and discussed in a number of editorials/commentaries (Strielkowski, 2018; Grudniewicz et al., 2019). Journals that do not adhere to the reputable peer review codes and that have no societal or academic affiliations. Whether the reason for this trend is fueled solely by profit or also by pressure to publish for promotion and advancement is unimportant when you consider the increases in the likelihood that unreliable and non-reproduceable data are entering the scientific and public domain. Paralleling the proliferation of journals is the surge in invitations we receive to serve on editorial boards and be associate editors for journals beyond our qualifications and to attend scientific meetings without merit or the backing of any legitimate scientific society or academic organization. These trends not only threaten the integrity of our field but they are dangerous when one considers that this can unduly influence the lay public and government. Collectively, in our opinion these trends do not facilitate the advancement of science.

Also worthy of further comment is that over the last decade the biomedical research community has increasingly questioned why published studies are often difficult to replicate. Sometimes even within the same laboratory. Importantly this has prompted increased attention being given to the fidelity and source of reagents (e.g., specificity of antibodies, genetic constructs) and experimental models (genetically engineered animals and cells for tissue culture). While this topic has now been the subject of many editorials and this issue is being embraced by funding agencies (for example, see NIH guidelines for rigor and reproducibility www.nih.gov/research-training/rigor-reproducibility), reputable journals, discussion boards and individual scientists it is an issue that will require continued consideration by the community and by the individual scientist.

Reviewing the last 10 years of Frontiers in Vascular Physiology it has been interesting to examine/study the trends in the field. Some insights can be seen by looking at published topics that have received the most attention in terms of downloads and citations. These are summarized in the following tables (Table 1). Importantly, the Frontiers in Vascular Physiology is publishing studies that are contributing to our knowledge of vascular biology spanning basic, translational and therapeutic topics. Further, our publications reflect the global community of vascular physiologists. With the advances in vascular physiology anticipated for the next decade the future of the Journal seems equally promising and will continue to be a forum for us all to witness the continued progress of the field.

Table 1Top ten articles downloads (upper table) and citations (lower table) from Frontiers in Vascular Physiology in the last decade.

**Table:** 

**Title**	**DOI**	**Views**	**PDF Downloads**
MicroRNA regulation of SIRT1	10.3389/fphys.2012.00068	11,878	5,207
Role of tumor associated macrophages in tumor angiogenesis and lymphangiogenesis	10.3389/fphys.2014.00075	16,268	5,207
The CXCL12/CXCR4 chemokine ligand/receptor axis in cardiovascular disease	10.3389/fphys.2014.00212	20,178	4,967
Transforming growth factor-β and endoglin signaling orchestrate wound healing	10.3389/fphys.2011.00089	15,844	4,860
Caveolae, caveolins, cavins, and endothelial cell function: new insights	10.3389/fphys.2011.00120	11,259	4,547
Effects of exercise on cardiovascular performance in the elderly	10.3389/fphys.2014.00051	24,669	4,114
Macrophage phenotype modulation by CXCL4 in atherosclerosis	10.3389/fphys.2012.00001	12,129	3,897
Vascular inflammatory cells in hypertension	10.3389/fphys.2012.00128	11,986	3,871
Mesenchymal stem cell-derived extracellular vesicles promote angiogenesis: potential clinical application	10.3389/fphys.2016.00024	12,319	3,776
Molecular pathways of notch signaling in vascular smooth muscle cells	10.3389/fphys.2012.00081	10,721	3,578

**Table:** 

**Title**	**DOI**	**Views**
Role of tumor associated macrophages in tumorangiogenesis and lymphangiogenesis	10.3389/fphys.2014.00075	16,268
Caveolae, caveolins, cavins, and endothelial cell function: new insights	10.3389/fphys.2011.00120	11,259
Mechanisms involved in the aging-induced vascular dysfunction	10.3389/fphys.2012.00132	8,830
The CXCL12/CXCR4 chemokine ligand/receptor axis in cardiovascular disease	10.3389/fphys.2014.00212	2,017
Vascular inflammatory cells in hypertension	10.3389/fphys.2012.00128	11,986
The role IL-1 in tumor-mediated angiogenesis	10.3389/fphys.2014.00114	9,407
MicroRNA regulation of SIRT	110.3389/fphys.2012.00068	11,878
Vulnerability of the developing brain to hypoxic-ischemic damage: contribution of the cerebral vasculature to injury and repair?	10.3389/fphys.2012.00424	13,838
Mesenchymal stem cell-derived extracellular vesicles promote angiogenesis: potential clinical application	10.3389/fphys.2016.00024	12,319
Macrophages and chemokines as mediators of angiogenesis	10.3389/fphys.2013.00159	10,289

## Author Contributions

All authors listed have made a substantial, direct and intellectual contribution to the work, and approved it for publication.

## Conflict of Interest

The authors declare that the research was conducted in the absence of any commercial or financial relationships that could be construed as a potential conflict of interest.
